# Gender differences in the incidence, characteristics and hospital admission outcomes of fall-related injuries in older adults in Victoria, Australia, over 5 years from 2018/19 to 2022/23

**DOI:** 10.3389/fpubh.2024.1426726

**Published:** 2024-11-14

**Authors:** Janneke Berecki-Gisolf, Ehsan Rezaei-Darzi, Aleksandra Helena Natora

**Affiliations:** ^1^Victorian Injury Surveillance Unit, Monash University Accident Research Centre, Monash University, Melbourne, VIC, Australia; ^2^Public Health Prevention Programs, Community and Public Health Division, Department of Health, State Government of Victoria, Melbourne, VIC, Australia

**Keywords:** injury epidemiology, fall-related injury, gender, sex, hospital outcomes, injury prevention

## Abstract

**Background:**

Falls are the leading cause of injury morbidity and mortality in older adults. This study aimed to: (1) Explore gender differences in falls injury incidence and outcomes in Victoria, Australia; and (2) Test if these differences are explained by patient demographics and clinical complexity.

**Method:**

Fall-related injury admissions records from 1-JULY-2018 to 30-JUNE-2023 were extracted from the Victorian Admitted Episodes Dataset. Admissions for injury (S00-T98) caused by a fall (W00-W19), in males and females aged 60+ years, were selected using ICD-10-AM codes. Incidence was calculated as annual falls admissions per 100,000 population. Gender differences in terms of demographics, falls details, injury types, complexity and admission outcomes were tested using logistic regression models.

**Results:**

There were 187,878 fall-related injury admissions: 67,635 (36.0%) by males and 120,243 (64.0%) by females. The incidence rate ratio peaked at 1.52 (female: male) at 70–79 years. Compared to males, female fall injuries were more likely due to same-level falls and to occur at home. Female sex was associated with fractures and male sex was associated with head injuries. Although female sex was associated with surgery and longer hospital stay, death-in-hospital was associated with male sex, with and without adjustment for patient demographics, fall details, injury type and clinical complexity.

**Conclusion:**

This contemporary gender-stratified study provides important evidence relevant to falls prevention and management. The findings suggest that same-level falls prevention is of particular relevance to females while in males, improved hospital outcomes and fall-related injury survivability, and any underlying frailty, should be prioritized.

## Introduction

Falls and falls-related injury among older people is an increasing global health issue (1). Falls are the leading cause of injury-related morbidity and mortality among older adults over 65 years of age (2), and result in more years lived with disability than transport injury, poisoning, drowning and burns combined (3). Falls are tremendously costly to the health, safety and wellbeing of older adults, as well as to health and aged care systems worldwide (4–8).

International epidemiology of falls indicates one in three older adults fall each year, with over 10% having multiple falls each year (9, 10). The frequency of falls increases with age and frailty (1). Falls result in injury for around 22 to 60% of cases, and of these it is estimated 20% sustain injuries requiring hospitalization (11).

Gender and sex differences are commonly reported in international falls literature with respect to risk factors, incidence, characteristics and outcomes of falls among older adults (12). It is worth noting that the definition of older adults can vary between studies: although commonly defined as 65+ years, in the current study, the focus is on adults aged 60+ years.

Consistent findings indicate that older females have a higher risk and incidence of falls than older males (13). Females sustain more severe fall-related injuries and are more likely to be hospitalized than males (14), while males are more likely to die as a result of falls (15–17). Australian data also indicates that falls-related injury disproportionately affects older females above the age of 65 years (18). In 2019–20, older females had a larger proportion of falls-related injury hospital admissions (63%) and falls-related injury deaths (53%). Falls-related injury hospitalization rates were reported to be 26% higher for older females than males (3,571 per 100,000 vs. 2,629 per 100,000), while falls-related injury death rates were 19% higher for older males than females (128 per 100,00 vs. 103 per 100,000). Australian females experienced more frequent same-level falls and more fractures than males, while males experienced more falls-related head and intracranial injuries than females (18, 19).

Gender disparities in falls-related injury highlight that older adults are frequent users of hospital services and that males and females have distinct healthcare and other support needs. Governments worldwide have responded and formulated public health policies to prevent falls and reduce their burden on individuals and health and aged care systems (20). International, evidence-based guidelines are now also well-established for the prevention and management of falls among older adults (3, 21). However, these policies and guidelines provide little specific guidance on gender stratification for falls prevention among older adults, though they often acknowledge fall-related injuries need to be managed in light of potential gender differences in underlying medical conditions associated with an increased risk of falls. Several studies suggest the need for better targeting of falls prevention strategies for males and females, respectively, (22–25). Indeed, contemporary public health points to the need for greater understanding of gender-related disparities and impacts of falls. This will ensure gender-responsive health service provision and clinical care, and gender-equitable falls prevention policies and programs to achieve improved health outcomes for older adults.

Therefore, this study aims to determine gender differences in the incidence, injury circumstances and hospital treatment outcomes of falls-related injuries among older adults in Victoria, Australia. The following research questions are addressed: (1) How do males and females differ in their rates of hospital-admitted falls? (2) How do males and females differ in their fall-related injury type, cause and outcomes? (3) Are female/male differences in falls outcomes explained by differences in age and injury type?

## Methods

This epidemiological study of fall-related injuries in Victoria, Australia is based on secondary analysis of administrative hospital admissions records. These data are combined with residential population estimates to calculate fall-related injury incidence in males and females, and analyzed in terms of sociodemographic factors, other patient characteristics and injury complexity to determine gender differences in fall-related injury types and severity.

### Data sources

The Victorian hospital admissions data used in this study were derived from the Victorian Admitted Episodes Dataset (VAED) which was provided to the Victorian Injury Surveillance Unit by the Victorian Department of Health. It contains latest available information on patient demographics such as age, gender, and residential location; clinical details such as primary and secondary diagnoses using International Classification of Diseases (ICD-10-AM) codes; procedures performed during the hospital stay coded with Australian Classification of Health Interventions (ACHI) codes, and the length of stay in the hospital. It also contains information on admission type (e.g., emergency, elective), discharge status, and admission source. Australian Bureau of Statistics (ABS) residential population estimates for Victoria, Australia, 2018/19–2022/23, were used to calculate population based rates (26).

### Sample selection

Fall-related injury cases were selected as injury admissions, based on the first-listed diagnosis code, caused by a fall, based on the first-listed external cause code. Injury diagnoses were selected as ICD-10-AM codes in the range of S00-T98 and falls were selected as ICD-10-AM codes in the range of W00-W19. Only cases admitted between 1 July 2018 and 30 June 2023 were included: a study period of five financial years. To prevent overcounting, only incident admissions were selected, by excluding statistical separations (internal transfers within the same hospital) and transfers (to another hospital) and excluding admissions that were flagged as repeat treatment. Only patients that were residents of Victoria, Australia and aged 60 years or above were included. The sample was limited to male or female patients only, as other patients recorded as ‘indeterminate’ or ‘other’ sex were excluded due to small numbers which would compromise privacy and confidentiality.

### Variables

#### Patient demographics

Age was considered in 5-year age bands from 60 to 64 years through to 85+ years. Gender was defined as a person’s sex of male or female, derived from the ‘sex’ data variable in the VAED. Victorian health services are required to report a patient’s sex in the VAED in accordance with Australian Bureau of Statistics Standard for Sex, Gender, Variations of Sex Characteristics and Sexual Orientation Variables (27). Please note, this study includes data for years *prior* to new reporting requirements for the VAED to include data for ‘sex at birth’ rather than ‘sex’, and ‘gender’, effective from 1 July 2024.

#### Falls details

Injury cause was based on the falls ICD-10-AM codes and grouped as falls on the same level (W01, W03, W18), falls from furniture (W06-W08), falls from stairs or steps (W10), falls from height/other level (W11-13, W16-17), other falls (W00, W02, W04-05, W09, W14-15), and unspecified falls (W19). Place and activity when injured were based on the relevant ICD-10-AM codes (Y92 and U73, respectively).

#### Injury type

The injury type and bodily location were based on the ICD-10-AM first-recorded injury diagnosis code (the principal injury) and categorized into broad groups. Infrequently occurring injury types were grouped together; these groupings are specified in table footnotes.

#### Case complexity and admission outcome

Comorbidity was captured using the Australian Injury Comorbidity Index (AICI) for mortality and grouped as 0, 1–2 and 3+ conditions (28). Length of stay was captured by combining the field indicating same-day, overnight, multiday stay and the total duration in days, to create a variable indicating LOS as same-day, overnight, 3–7 days or longer than 1 week. Surgery was determined from the Diagnosis Related Group (DRG) variable in the VAED: the second and third characters of the DRG are numerical and were used to identify the grouping. Cases where this number was in the range of 01 to 39 were grouped as surgical admissions. Discharge destination, which includes death, discharge to usual home residence, and various transfer destinations, was based on the corresponding data field in the VAED.

### Data analysis

All analyses were gender stratified. Descriptive statistics are shown for males and females; demographic descriptive statistics are given as frequency and population-based rate whereas all other descriptive statistics are shown as frequency and percentage only. Frequencies and population-based rates are based on all selected incident admissions: if the same person has multiple *incident* fall-related injury admissions (not including transfers and repeat treatments), these are all included as separate counts. Population-based falls rates were calculated as annual fall-related injury admissions per 1,000 population [based in residential population estimates (26)], by sex, age group and financial year. Female/male incidence rate ratios (IRRs) and 95% confidence intervals (CI) were calculated. Descriptive statistics of sex differences in fall circumstances, injury type and clinical characteristics/outcome were compared using chi-square testing; the resulting *p*-values are reported. Among fall-related injury admissions, gender differences in patient characteristics, falls circumstances, case complexity and hospital outcomes were explored using logistic regression models and reported as odds ratios (OR) with 95% CI. The dependent variable in these models was gender: female vs. male (which was the reference category). All variables were first explored in univariable models, followed by a series of multivariate models in the following sequence:

1. Patient demographics
2. Patient demographics + fall cause and circumstances
3. Patient demographics + fall cause and circumstances + injury type
4. Patient demographics + fall cause and circumstances + injury type + clinical characteristics/ outcomes

Analyses were conducted using SAS Vs. 9.4 software.

### Ethics statement

The Victorian Injury Surveillance Unit has Monash University Human Research Ethical Committee (MUHREC) approval for the analysis of the VAED for generating Victorian injury statistics (MUHREC project ID: 21427).

## Results

In the five-year period from 1 July 2018 and 30 June 2023 there were 187,878 hospital admissions for fall-related injuries among people aged 60 years and above in Victoria: 67,635 (36.0%) by males and 120,243 (64.0%) by females.

The most commonly occurring principal injury diagnoses among males and females are listed in Table 1: the 20 most common diagnoses account 84.8% of all cases in males and 88.3% in females. Femur fracture was the most common injury in both males and females. Head injuries were proportionally more common in males, with open head wounds, superficial head injury, intracranial injury, skull/facial bone fracture and other and unspecified head injuries together accounting for 30.2% of injuries in males and 23.1% in females. Most types of fractures were proportionally more common in females; the most notable being forearm fracture which accounted for 9.5% of fall-related injuries in females and 2.8% in males. Rib/sternum/thoracic spine fractures were an exception: these were more common in males (10.8%) than females (6.5%).

**Table 1:** Fall-related injury in males and females: 20 most commonly occurring principal diagnoses, Victoria, Australia, 2018/19 to 2022/23.

	Females		Males	
#	Principal diagnosis	*N* (%)	Principal diagnosis	*N* (%)
1	Fracture of femur	18,366 (15.3)	Fracture of femur	8,263 (12.2)
2	Fracture of forearm	11,393 (9.5)	Open wound of head	7,376 (10.9)
3	Fracture of shoulder and upper arm	8,942 (7.4)	Fracture of rib(s), sternum and thoracic spine	7,283 (10.8)
4	Open wound of head	8,936 (7.4)	Intracranial injury	5,294 (7.8)
5	Fracture of lower leg, including ankle	7,964 (6.6)	Fracture of lumbar spine and pelvis	3,661 (5.4)
6	Fracture of rib(s), sternum and thoracic spine	7,794 (6.5)	Superficial injury of head	3,565 (5.3)
7	Fracture of lumbar spine and pelvis	7,706 (6.4)	Fracture of lower leg, including ankle	2,803 (4.1)
8	Superficial injury of head	7,037 (5.9)	Fracture of shoulder and upper arm	2,759 (4.1)
9	Intracranial injury	5,138 (4.3)	Other and unspecified injuries of head	2,359 (3.5)
10	Other and unspecified injuries of head	3,714 (3.1)	Fracture of forearm	1,902 (2.8)
11	Other and specified injuries of hip and thigh	3,251 (2.7)	Fracture of skull and facial bones	1,884 (2.8)
12	Other and unspecified injuries of abdomen, lower back and pelvis	2,869 (2.4)	Other and unspecified injuries of abdomen, lower back and pelvis	1,735 (2.6)
13	Fracture of skull and facial bones	2,851 (2.4)	Other and specified injuries of hip and thigh	1,615 (2.4)
14	Open wound of lower leg	1,825 (1.5)	Open wound of forearm	1,372 (2)
15	Other and unspecified injuries of lower leg	1,744 (1.5)	Other and unspecified injuries of thorax	1,162 (1.7)
16	Other and unspecified injuries of thorax	1,387 (1.2)	Fracture of neck	1,048 (1.5)
17	Superficial injury of lower leg	1,366 (1.1)	Other and unspecified injuries of lower leg	9,09 (1.3)
18	Other and unspecified injuries of shoulder and upper arm	1,359 (1.1)	Other and unspecified injuries of shoulder and upper arm	8,37 (1.2)
19	Fracture at wrist and hand level	1,317 (1.1)	Open wound of lower leg	7,62 (1.1)
20	Dislocation, sprain and strain of joints and ligaments of shoulder girdle	1,219 (1)	Fracture at wrist and hand level	7,59 (1.1)
Total		106,178 (88.3)		57,348 (84.8)

**Figure 1:**
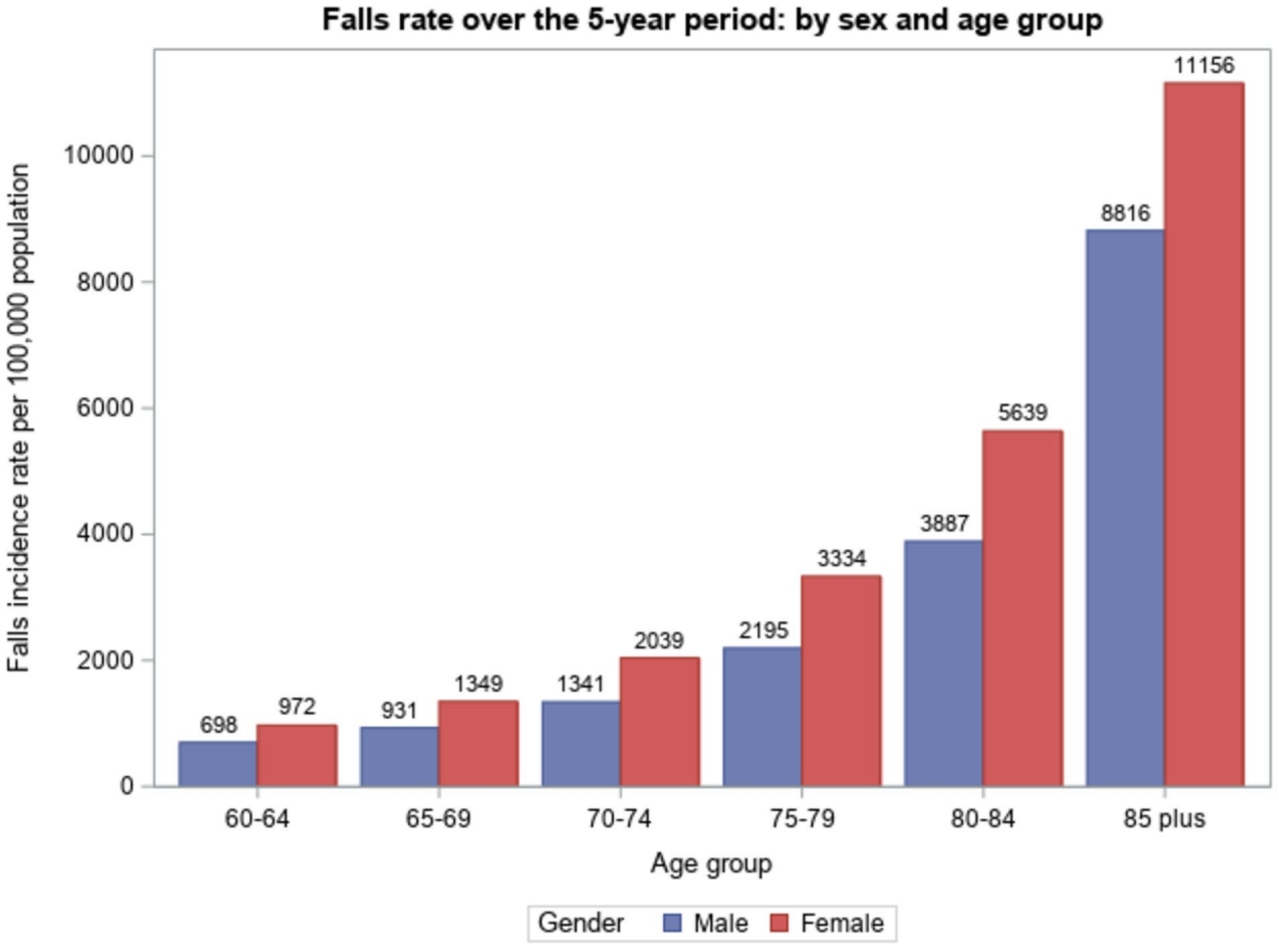
Hospital-admitted fall-related injury incidence per 100,000 population over the 5-year period: by sex and age group, Victoria, Australia, 2018/19 to 2022/23.

Fall-related injury admission rates for males and females are shown in Figure 1 and Table 2. The oldest age group, 85+ years, accounted for the greatest proportion of fall-related injury admissions in both males (35.0%) and females (39.3%). Fall-related injury admission rates increased steeply with increasing age for both males and females. From age 60–64 years to 85+ years, rates per 100,000 population increased incrementally from 689 to 8,816 in males and from 972 to 11,156 in females. In each age group, fall-related injury admission rates were higher in females than males: the rate ratio ranged from a minimum of 1.27 in the 85+ years age group to a maximum of 1.52 in the 70–74 and 75–79 year age groups. The gender ratio changed over the five-year period, gradually decreasing from a rate ratio of 1.64 in 2018/19 to 1.51 in 2022/23.

**Table 2:** Patient demographics of fall-related injury: sex-stratified population-based incidence by age group and year of admission in Victoria, 2018/19 to 2022/23.

Demographic characteristics	Females		Males		Rate Ratio F *vs* M	
	*N* (%)	Incidence per 100,000 population	*N* (%)	Incidence per 100,000 population	RR	[95% CI]
Age group						
60–64	8,992 (7.5)	972	6,020 (8.9)	698	1.39	[1.35, 1.44]
65–69	11,009 (9.2)	1,349	6,963 (10.3)	931	1.45	[1.41, 1.49]
70–74	14,509 (12.1)	2,039	8,721 (12.9)	1,341	1.52	[1.48, 1.56]
75–79	17,339 (14.4)	3,334	10,268 (15.2)	2,195	1.52	[1.48, 1.56]
80–84	21,107 (17.6)	5,639	12,017 (17.8)	3,887	1.45	[1.42, 1.48]
85+	47,287 (39.3)	11,156	23,646 (35.0)	8,816	1.27	[1.25, 1.29]
Year						
2018/19	23,720 (19.7)	3,330	12,701 (18.8)	2,028	1.64	[1.61, 1.68]
2019/20	23,768 (19.8)	3,221	13,141 (19.4)	2,030	1.59	[1.55, 1.62]
2020/21	24,168 (20.1)	3,190	13,869 (20.5)	2,091	1.53	[1.49, 1.56]
2021/22	24,078 (20)	3,118	13,708 (20.3)	2,027	1.54	[1.51, 1.57]
2022/23	24,509 (20.4)	3,098	14,216 (21)	2,053	1.51	[1.48, 1.54]

Table 3 shows the fall-related injury admission frequency breakdown by falls circumstances, injury type, clinical characteristics and outcomes. Same-level falls were relatively common in females whereas falls from height were relatively common in males. Home was the most common place of fall occurrence in males and females. The activity when injured was unspecified in the majority of cases, in males as well as females (69.5% vs. 71.1%, respectively). Male sex was more commonly associated with work-related activities, while vital activity (resting eating sleeping) was slightly more common among females. The most common injury type was fractures: these accounted for 57.0% of injuries in females and 45.6% in males. Intracranial injuries and open wounds were relatively common in males. In males, the location of the injury was relatively likely to be head/face/neck or trunk, while lower or upper extremity injuries were relatively more common in females. Males were more likely to have any, or multiple comorbidities. The length of stay was similar in males and females, but females were more likely to have surgery during their hospital stay. Death in hospital was more likely in males than females (2.4% vs. 1.3%, respectively). The most common discharge destination was to the patient’s home for both males and females.

**Table 3:** Descriptive statistics (frequency and percentage, with chi-square testing) of sex differences in fall circumstances, injury type and clinical characteristics/outcome, Victoria, Australia, 2018/19 to 2022/23.

	Females	Males	Chi-square, *p*-value
	*N* (%)	*N* (%)	
Injury cause			
Falls on the same level	68,696 (57.1)	34,154 (50.5)	<0.0001
Falls from furniture	7,555 (6.3)	4,596 (6.8)	
Falls from stairs or steps	7,422 (6.2)	3,958 (5.9)	
Falls from height/other level	2,461 (2.0)	5,533 (8.2)	
Other falls	1,197 (1.0)	1,129 (1.7)	
Unspecified falls	32,912 (27.4)	18,265 (27.0)	
Place of occurrence			<0.0001
Home	61,394 (51.1)	33,257 (49.2)	
Residential Institution	22,500 (18.7)	11,037 (16.3)	
School, public buildings	2,003 (1.7)	1,154 (1.7)	
Sports & athletic areas	540 (0.4)	444 (0.7)	
Road, street & highway	5,303 (4.4)	3,336 (4.9)	
Trade & service area	3,741 (3.1)	2,013 (3)	
Industrial & construction area	43 (0.0)	197 (0.3)	
Farm	103 (0.1)	219 (0.3)	
Other specified places	2,827 (2.4)	1,654 (2.4)	
Unspecified places	21,789 (18.1)	14,324 (21.2)	
Activity when injured			<0.0001
Sports	2,040 (1.7)	1,252 (1.9)	
Leisure	2,116 (1.8)	1,123 (1.7)	
Working for income	661 (0.5)	1,003 (1.5)	
Other types of work-unpaid	9,381 (7.8)	6,006 (8.9)	
Vital activities, resting, eating, sleeping	15,066 (12.5)	8,187 (12.1)	
Other specified activity	5,509 (4.6)	3,048 (4.5)	
Unspecified activity	85,470 (71.1)	47,016 (69.5)	
Injury type			<0.0001
Superficial injury	11,041 (9.2)	6,253 (9.2)	
Dislocation, sprain and strain	3,546 (2.9)	1,971 (2.9)	
Fracture	68,486 (57.0)	30,845 (45.6)	
Eye injury- excl foreign body	490 (0.4)	210 (0.3)	
Injury to internal organs	347 (0.3)	526 (0.8)	
Injury to muscle and tendon	1,152 (1.0)	1,307 (1.9)	
Injury to nerves and spinal cord	141 (0.1)	207 (0.3)	
Intracranial injury	5,138 (4.3)	5,294 (7.8)	
Open wound	12,737 (10.6)	10,480 (15.5)	
Other^1^	102 (0.1)	110 (0.2)	
Other and unspecified injury	16,075 (13.4)	9,684 (14.3)	
Other effects of external cause/complications/late effects	988 (0.8)	748 (1.1)	
Bodily location of injury			<0.0001
Body region not relevant	1,014 (0.8)	763 (1.1)	
Head/face/neck	30,300 (25.2)	22,617 (33.4)	
Lower extremity	39,366 (32.7)	17,890 (26.5)	
Trunk	21,150 (17.6)	15,309 (22.6)	
Unspecified body region^2^	75 (0.1)	65 (0.1)	
Upper extremity	28,338 (23.6)	10,991 (16.3)	
Comorbidity			<0.0001
0	82,681 (68.8)	40,430 (59.8)	
1–2	35,154 (29.2)	24,748 (36.6)	
> = 3	2,408 (2.0)	2,457 (3.6)	
Length of stay			<0.0001
Same day	31,212 (26.0)	18,790 (27.8)	
Overnight	25,142 (20.9)	14,189 (21.0)	
3–7 days	39,804 (33.1)	21,326 (31.5)	
>1 week	24,085 (20.0)	13,330 (19.7)	
Surgery			<0.0001
Yes	30,059 (25.0)	137,48 (20.3)	
No	90,184 (75.0)	53,887 (79.7)	
Discharge destination			<0.0001
Mental health residential facility^3^	62 (0.1)	41 (0.1)	
Transition Care bed based program^3^	945 (0.8)	367 (0.5)	
Death in hospital	1,569 (1.3)	1,631 (2.4)	
Private residence/accommodation	67,549 (56.2)	40,097 (59.3)	
Residential aged care facility, which is not the usual place of residence^3,4^	1,099 (0.9)	583 (0.9)	
Residential aged care facility, which is the usual place of residence^3,4^	4,928 (4.1)	2,438 (3.6)	
Aged care residential facility^3,4^	9,194 (7.6)	4,476 (6.6)	
Statistical Separation^3^	6,473 (5.4)	3,240 (4.8)	
Acute hospital/extended care^3^	27,930 (23.2)	14,214 (21.0)	
Left against medical advice	494 (0.4)	548 (0.8)	
*Total*	*120,243 (100)*	*67,635 (100)*	

^1injury to blood vessels, crushing injury, traumatic amputation, foreign body, burns, systemic-poisoning/toxic effects, Complications of surgical and med care NEC; 2Includes a few Missing injury code cases for females only; 3Separation and transfer; 4Aged care residential facility was used until 2021/22 after which it was broken down into two categories, based on whether it was the usual place of residence or not.^

Univariate and multivariable logistic regression results of factors associated with female vs. male hospital-admitted fall-related injuries are shown in Table 4. In the univariate as well as fully adjusted modelling, female (vs. male) fall injury admissions were associated with older age (85+ years) and occurrence in 2018/19 to 2019/20 compared to more recent years. Female vs. male fall injuries were more likely to be caused by same-level falls. In females, place of occurrence was more likely to be home or residential institution and less likely to be industrial and construction area. In females, activity when injured was less likely to be working for income. Female sex was associated with fractures, as evidenced from the low odds ratios in all other injury types relative to fractures. Female sex was associated with upper and lower extremity injuries, whereas head/face/neck and trunk injuries were negatively associated with female sex. Females were less likely than males to have any, or multiple, recorded comorbidities. Females (vs. males) were more likely to have overnight or longer hospital stays. In the fully adjusted model, there were no statistically significant sex differences in surgery, but female sex was associated with surgery in the univariate analysis. Female sex was negatively associated with death in hospital, in univariate as well as fully adjusted modelling.

**Table 4:** Logistic regression models (univariate and multivariable) of sex differences in fall injury demographics (model 1) with fall circumstances (model 2), injury type (model 3) and clinical characteristics/outcome (model 4), Victoria, Australia, 2018/19 to 2022/23.

	Univariate	Model 1	Model 2	Model 3	Model 4
	OR [95% CI]	OR [95% CI]	OR [95% CI]	OR [95% CI]	OR [95% CI]
Age group					
60–64	0.89 [0.85,0.92]	0.88 [0.85,0.92]	1.04 [1.00,1.09]	0.98 [0.94,1.02]	0.94 [0.90,0.98]
65–69	0.94 [0.90,0.97]	0.94 [0.90,0.97]	1.04 [1.00,1.09]	0.99 [0.95,1.03]	0.96 [0.92,1.00]
70–74	0.99 [0.95,1.02]	0.99 [0.95,1.02]	1.03 [0.99,1.07]	1.00 [0.97,1.04]	0.99 [0.96,1.03]
75–79	Reference	Reference	Reference	Reference	Reference
80–84	1.04 [1.01,1.08]	1.04 [1.01,1.07]	1.00 [0.97,1.04]	1.02 [0.98,1.05]	1.03 [0.99,1.06]
85+	1.18 [1.15,1.22]	1.18 [1.15,1.22]	1.11 [1.08,1.14]	1.15 [1.11,1.18]	1.15 [1.11,1.18]
Year					
2018/19	1.08 [1.05,1.12]	1.08 [1.05,1.11]	1.08 [1.05,1.12]	1.08 [1.05,1.11]	1.08 [1.04,1.11]
2019/20	1.05 [1.02,1.08]	1.05 [1.02,1.08]	1.05 [1.02,1.08]	1.05 [1.01,1.08]	1.04 [1.01,1.07]
2020/21	1.01 [0.98,1.04]	1.01 [0.98,1.04]	1.01 [0.98,1.04]	1.01 [0.98,1.04]	1.01 [0.98,1.05]
2021/22	1.02 [0.99,1.05]	1.02 [0.99,1.05]	1.01 [0.99,1.05]	1.01 [0.98,1.04]	1.01 [0.98,1.04]
2022/23	Reference	Reference	Reference	Reference	Reference
Injury cause					
Falls on the same level	Reference		Reference	Reference	Reference
Falls from furniture	0.82 [0.79,0.85]		0.79 [0.76,0.82]	0.83 [0.8,0.86]	0.84 [0.81,0.88]
Falls from stairs or steps	0.93 [0.90,0.97]		0.95 [0.91,0.99]	0.95 [0.91,0.99]	0.93 [0.89,0.97]
Falls from height/other level	0.22 [0.21,0.23]		0.24 [0.22,0.25]	0.25 [0.23,0.26]	0.23 [0.22,0.25]
Other falls	0.53 [0.49,0.57]		0.54 [0.49,0.58]	0.56 [0.52,0.61]	0.58 [0.53,0.63]
Unspecified falls	0.90 [0.88,0.92]		0.88 [0.85,0.90]	0.90 [0.88,0.93]	0.92 [0.90,0.94]
Place of occurrence					
Home	Reference		Reference	Reference	Reference
Residential Institution	1.10 [1.08,1.13]		1.07 [1.04,1.10]	1.09 [1.06,1.12]	1.06 [1.03,1.10]
School, public buildings	0.94 [0.87,1.01]		0.95 [0.88,1.03]	0.97 [0.89,1.04]	0.98 [0.91,1.06]
Sports and athletic areas	0.66 [0.58,0.75]		0.64 [0.56,0.73]	0.63 [0.54,0.72]	0.58 [0.51,0.67]
Road, street and highway	0.86 [0.82,0.90]		0.82 [0.78,0.86]	0.80 [0.77,0.84]	0.77 [0.73,0.80]
Trade and service area	1.01 [0.95,1.06]		0.98 [0.93,1.04]	0.98 [0.93,1.04]	0.96 [0.91,1.02]
Industrial and construction area	0.12 [0.09,0.17]		0.26 [0.18,0.36]	0.26 [0.18,0.37]	0.26 [0.18,0.37]
Farm	0.26 [0.20,0.32]		0.34 [0.27,0.43]	0.33 [0.25,0.42]	0.3 [0.24,0.39]
Other specified places	0.93 [0.87,0.99]		0.94 [0.88,1.00]	0.91 [0.86,0.98]	0.87 [0.81,0.93]
Unspecified places	0.82 [0.80,0.85]		0.89 [0.87,0.91]	0.87 [0.84,0.89]	0.84 [0.81,0.86]
Activity when injured					
Sports	0.89 [0.82,0.96]		1.13 [1.04,1.22]	1.02 [0.94,1.11]	0.96 [0.88,1.05]
Leisure	1.02 [0.95,1.11]		1.14 [1.06,1.24]	1.08 [1.00,1.18]	1.04 [0.96,1.13]
Working for income	0.36 [0.32,0.40]		0.62 [0.55,0.69]	0.59 [0.53,0.67]	0.56 [0.50,0.63]
Other types of work-unpaid	0.85 [0.81,0.89]		1.08 [1.04,1.13]	1.03 [0.99,1.08]	0.98 [0.94,1.03]
Vital activities, resting, eating, sleeping	Reference		Reference	Reference	Reference
Other specified activity	0.98 [0.93,1.03]		1.08 [1.03,1.14]	1.05 [0.99,1.11]	1.02 [0.97,1.08]
Unspecified activity	0.99 [0.96,1.02]		1.07 [1.04,1.11]	1.05 [1.02,1.08]	1.04 [1.00,1.07]
Injury type					
Superficial injury	0.80 [0.77,0.82]			0.81 [0.78,0.84]	0.81 [0.77,0.84]
Dislocation, sprain and strain	0.81 [0.77,0.86]			0.69 [0.65,0.74]	0.67 [0.63,0.71]
Fracture	Reference			Reference	Reference
Eye injury- excl foreign body	1.05 [0.89,1.24]			1.13 [0.95,1.33]	1.12 [0.95,1.33]
Injury to internal organs	0.30 [0.26,0.34]			0.47 [0.41,0.54]	0.48 [0.42,0.56]
Injury to muscle and tendon	0.40 [0.37,0.43]			0.35 [0.32,0.38]	0.33 [0.31,0.36]
Injury to nerves and spinal cord	0.31 [0.25,0.38]			0.39 [0.31,0.48]	0.40 [0.32,0.50]
Intracranial injury	0.44 [0.42,0.46]			0.49 [0.47,0.51]	0.52 [0.50,0.55]
Open wound	0.55 [0.53,0.56]			0.53 [0.51,0.54]	0.51 [0.49,0.53]
Other^1^	0.42 [0.32,0.55]			0.39 [0.28,0.53]	0.40 [0.29,0.54]
Other and unspecified injury	0.75 [0.73,0.77]			0.78 [0.75,0.80]	0.77 [0.75,0.80]
Other effects of external cause/complications/late effects	0.60 [0.54,0.66]			0.29 [0.14,0.58]	0.36 [0.18,0.74]
Bodily location of injury					
Body region not relevant	0.60 [0.55,0.67]			1.74 [0.85,3.53]	1.48 [0.73,3.03]
Head/face/neck	0.61 [0.59,0.62]			0.80 [0.78,0.83]	0.77 [0.75,0.80]
Lower extremity	Reference			Reference	Reference
Trunk	0.63 [0.61,0.65]			0.63 [0.61,0.65]	0.62 [0.60,0.64]
Unspecified body region	0.52 [0.38,0.73]			0.72 [0.51,1.01]	0.71 [0.50,1.00]
Upper extremity	1.17 [1.14,1.21]			1.26 [1.23,1.30]	1.20 [1.16,1.23]
Comorbidity					
0	Reference				Reference
1–2	0.70 [0.68,0.71]				0.64 [0.63,0.66]
> = 3	0.48 [0.45,0.51]				0.41 [0.39,0.44]
Length of stay					
Same day	Reference				Reference
Overnight	1.07 [1.04,1.10]				1.07 [1.04,1.10]
3–7 days	1.12 [1.10,1.15]				1.10 [1.07,1.13]
>1 week	1.09 [1.06,1.12]				1.07 [1.03,1.11]
Surgery					
Yes	1.31 [1.28,1.34]				1.01 [0.98,1.04]
No	Reference				Reference
Discharge destination					
Mental health residential facility*	0.90 [0.61,1.33]				0.85 [0.57,1.28]
Transition Care bed based program*	1.53 [1.35,1.73]				1.19 [1.05,1.36]
Death in hospital	0.57 [0.53,0.61]				0.59 [0.55,0.64]
Private residence/accommodation	Reference				Reference
Residential aged care facility, which is not the usual place of residence*†	1.12 [1.01,1.24]				1.04 [0.93,1.15]
Residential aged care facility, which is the usual place of residence*†	1.20 [1.14,1.26]				1.06 [1.00,1.12]
Aged care residential facility*†	1.22 [1.17,1.27]				1.06 [1.01,1.10]
Statistical Separation*	1.19 [1.14,1.24]				1.01 [0.97,1.06]
Acute hospital/extended care*	1.17 [1.14,1.19]				1.00 [0.97,1.02]
Left against medical advice	0.54 [0.47,0.61]				0.55 [0.49,0.63]

*Separation and transfer. †Aged care residential facility was used until 2021/22 after which it was broken down into two categories, based on whether it was the usual place of residence.

## Discussion

Using health service administrative data, this study quantified sex differences in falls circumstances, injury characteristics and clinical outcomes of fall-related injury hospitalizations among older Victorians aged 60+ years, from 2018/19 to 2022/23. The overall finding that falls-related injury disproportionately affected older females is broadly consistent with the vast majority of international literature on falls-related hospitalization (12). Females made up a greater proportion (64%) of hospitalized falls than males (36%), and females had higher rates of falls injury admission than males across all age-groups in our study. These findings confirm earlier Australian data (18, 20, 29), as well as international evidence from the US, Canada and England (15, 22, 30).

Falls injury admission rates increased steeply with increasing age among both males and females. This has historically been attributed to progressive loss of physical fitness, balance, mobility and muscle strength and increasing frailty with age in both sexes (1). However, when adjusting for comorbidities, lean and fat body mass and balance, a more recent study in Portugal found that actually males demonstrated higher risk of falling (31).

Falls injury hospital admission rates decreased more for females than males over the five-years in our study, as indicated by the gradually decreasing gender rate ratio from 1.64 in 2018/19 to 1.51 in 2022/23. This may reflect concerted efforts in screening and prevention of fractures (7, 19, 32) which we found to cause more hospital admissions among females than males.

The dominance of hospitalized falls-related fractures, particularly femur and hip fractures among females and head injuries among males, supports existing evidence (14, 18). This finding has previously been attributed to sex differences of reduced bone density and osteoporosis among post-menopausal females and loss of muscle strength and sarcopenia among males (33, 34). Sarcopenia was reported to be more strongly associated with balance deficits among males, while fat mass was more strongly associated with balance deficits in females (35).

Some of the gender disparity in falls injury causes we identified may reflect sex differences in levels and types of physical activity undertaken by older adults, which is an independent risk factor for falls (36). Australian physical activity data indicates males are slightly more physically active than females (37). The proportions of males and aged 65 and over who met physical activity guidelines were similar—27% for males and 23% for females. However, with increasing age, the proportion of males meeting the physical activity guideline remained the same, while for females the proportion decreased. For strength-based activity, 16% of females aged 65–74 years met the guidelines, compared with 8.1% of females aged 85 and over. This decrease was not seen in males, where the proportion completing at least 2 days of strength-based activities remained relatively similar for all age groups. This indicates a significant population level risk of falls. Encouraging participation in strength-based activities to reduce falls risk and improve mobility and stability can improve overall quality of life and reduce osteoporosis and sarcopenia (38) and lower the risk of falls and falls-related injury (39). More recent studies however highlight the need for gender-appropriate education and promotion of strength-based activities to optimize falls prevention among males and females, respectively (23, 25).

Another key finding of this study was the gender disparities in falls injury hospitalization outcomes showing higher rate of death in hospital among males compared to females. This falls-injury hospitalization outcome has previously been attributed to greater frailty and higher levels of comorbidities among males, while similarly or more frail older females endure long-term sequalae of falls-related injury. This has been referred to as the male–female health survival paradox (40–42) which suggests the need for more research to identify gender-responsive frailty interventions as part of falls prevention. We suggest the higher rate of death in hospital among males could also reflect prior undetected falls. Prior falls is known to be a significant risk factor for falls-related injury hospitalization. Males are less likely than females to report falls, seek medical care and/or discuss falls with health care providers (43), and this may contribute to their greater risk of death when they do come to experience falls-related hospitalization.

Living alone at the time of falls injury and availability of a carer at home to assist with activities of daily living and rehabilitation after fall hospitalization (44, 45) may explain why males were more likely to be discharged home more than females. Conversely, our finding that females were more likely than males to be discharged to longer-term hospital care programs or residential aged care was similar to another study that found females with comorbidities and longer stay in hospital have greater health service and aged care utilization (29). This may also reflect that females have greater fear of falling than males which can compromise their return home following falls hospitalization and reduce their independence (46, 47). However, a more recent study found fear of falling played a greater role as a predictor of self-care and household activities in males than in females (48). Reasons for gender disparities in falls-injury hospitalization discharge outcomes warrant further investigation.

The extent of gender differences in falls injury outcomes in our study was considerable. Female hospitalization rates for fall-related injuries were higher than in males, suggesting that females sustained more severe injuries. However, hospital admission *per se* is only a proxy indicator of injury severity (49). Severity is more accurately indicated by injury outcomes, such as the length of stay, comorbidity, if surgery was required, death in hospital, whether the patient was discharged to their usual home, or was (*de novo*) transferred to residential aged care (14). Our study found the rate ratios were higher among females for all these indicators, except for comorbidity, discharge home and death in hospital which were greater among males. The novel results of univariate and multi-variate logistic regression in our study provide important new information on gender disparities in falls injury outcomes. Clinical falls injury outcomes for females were indeed associated with older age, same-level types of falls, fractures and longer hospital stays. Male outcomes were associated with falls from height, work-related falls in industrial and construction areas, increased comorbidities and death in hospital.

The strengths of this study are its comprehensive, population-based approach, capturing all hospital-admitted fall-related injuries, state-wide. The focus on gender is another study strength: all results are fully stratified by gender, and the analysis is gender focused, whereas in most fall-related injury studies, gender is a covariate rather than a focus of the study. There are also study limitations that need to be acknowledged. First, this analysis is limited to hospital admitted injuries. These only constitute relatively severe injuries: non-admitted injuries are not captured. Potential gender differences in the decision to admit a patient, beyond factors directly related to the injury type and severity, are not addressed. It is recommended that further research address this gap through the analysis of non-admitted injury data, for example Emergency Department or general practice patient data. The decision to admit to hospital can be influenced by a range of factors (50, 51). In older adults, these factors can include the availability of care and support at home, and patient complexity (52). Gender differences in this decision making will affect patient case-mix in the hospital admission fall-related injury data, and further research is required to place the current study findings in this context. Second, the gender stratification is limited to males and females and does not capture other or undermined gender. The number of patients recorded in the VAED admissions data as of other or undetermined gender is insufficient to allow for the analysis presented here, without reporting small numbers that would compromise patient data confidentiality. It is recommended that future research utilizing larger datasets, for example national or international datasets spanning long periods of time, should address this. Such an approach may allow for analysis that is not underpowered or potentially compromising patient data confidentiality.

In conclusion, this injury surveillance study provides contemporary gender-stratified evidence on patterns of falls-related injuries and their hospitalization outcomes among older adults in Victoria, Australia. This information has important implications for progress in falls prevention and management and associated public health policy action in Victoria. The Victorian-specific information will provide useful learnings for other state and territory jurisdictions in Australia and may be applicable to many other countries with similarly ageing populations. This study recommends falls prevention to be actively included in broader public health policies that differentially target females’ and males’ health issues to achieve gender-equitable healthy ageing among older adults.

## Data Availability

The data analyzed in this study is subject to the following licenses/restrictions: this study was conducted in accordance with the data use agreement between the Victorian Injury Surveillance Unit and the Victorian Department of Health. The unit record data cannot be made available upon request by the authors to third parties as this would breach the data use agreement. Victorian Admitted Episodes Dataset (VAED) data can be requested directly from the Victorian Agency for Health Information at the Victorian Department of Health. Requests to access these datasets should be directed to Victorian Agency for Health Information, Victorian Department of Health; VAHI Data Request Hub, https://vahi.freshdesk.com/support/home.
